# Exploring the relationship between lipid-lowering drug target genes and sensorineural hearing loss by Mendelian randomization

**DOI:** 10.1097/MD.0000000000044174

**Published:** 2025-08-29

**Authors:** Yan Yang, Hao-Fei Huang, Kun-Lin Pu

**Affiliations:** a Department of Otorhinolaryngology, Pengzhou Hospital of Traditional Chinese Medicine, Chengdu, Sichuan, China.

**Keywords:** lipid-lowering drug, Mendelian randomization, sensorineural hearing loss

## Abstract

An increasing body of research indicates an association between lipid-lowering medications and sensorineural hearing loss (SNHL), although there is still controversy. Therefore, the aim of this study is to investigate the genetic correlation between different lipid-lowering therapeutic gene targets and SNHL. The genetic association between lipids, lipid-lowering drug target genes, and SNHL was analyzed using a 2-sample Mendelian randomization approach. The exposures included 5 circulating lipid levels (triglycerides, low-density lipoprotein cholesterol, high-density lipoprotein cholesterol, apolipoprotein A-I, and apolipoprotein B) and 10 target genes simulating the effects of lipid-lowering drugs (HMGCR, PCSK9, Niemann-Pick C1-like 1 [NPC1L1], LDLR, APOB, CETP, LPL, ANGPTL3, APOC3, and PPARA). Summary data from a large-scale genome-wide association study on SNHL from the Finnish database were used as the outcome. The inverse variance-weighted method was employed as the primary approach, with sensitivity tests conducted to evaluate heterogeneity and pleiotropy in the results. The genetic prediction of lipid levels was not significantly associated with SSNL. However, genetic proxies for lowering low-density lipoprotein cholesterol, specifically variants in NPC1L1 (OR = 1.943 [95% CI 1.116–3.383]; *P* = .018) and LDL receptor (LDLR) (OR = 1.279 [95% CI 1.107–1.477]; *P* < .001), were associated with an increased risk of SNHL. Similarly, a genetic proxy for lowering triglycerides, the apoprotein C-III (APOC3) variant (OR = 1.174 [95% CI 1.054–1.307]; *P* = .003), was associated with an increased risk of SNHL. After Bonferroni correction, the genetic variants for LDLR and APOC3 remained significantly associated with an increased risk of SNHL, while the association with the NPC1L1 lipid-lowering variant was no longer significant. This study suggests that lipid-lowering medications potentially have a causal impact on increasing the risk of SNHL through the LDLR and APOC3 pathways. LDLR and APOC3 show potential as candidate drug targets for the prevention of SNHL. However, the results of the study and the potential mechanism of action require further experimental validation and evaluation.

## 1. Introduction

Hearing loss is the most common sensory impairment in humans, typically resulting from damage to the sensory hair cells and primary auditory neurons caused by cochlear disease or trauma.^[1,2]^ More than 1.5 billion people worldwide experience hearing loss during their lifetime, with an estimated 466 million individuals globally affected by disabling hearing loss.^[3]^ The cost of hearing loss exceeds $980 billion annually worldwide, and if left untreated, it can result in a decrease in the quality of life at different stages of human life.^[4,5]^ Sensorineural hearing loss (SNHL) is the most common type of hearing loss.^[6]^ Its pathogenesis is not yet fully understood, and the etiology is complex and diverse, including infection, exposure to excessive noise, ototoxic drugs, or aging. In addition, obesity and hyperlipidemia may also be associated with hearing loss.^[1,7,8]^

Although hearing loss imposes a multifaceted burden, the prospect of preventing or treating hearing loss with medication remains elusive. Hearing loss is believed to be associated with inner ear microcirculation disorders. Previous observational studies have suggested an association between lipid levels and SNHL, indicating that elevated levels of low-density lipoprotein cholesterol (LDL-C) and triglycerides (TG) may exacerbate SNHL.^[8,9]^ Statins are a commonly used class of lipid-lowering drugs that reduce circulating LDL-C levels by targeting 3-hydroxy-3-methylglutaryl-coenzyme A reductase (HMGCR). Numerous studies have confirmed that the use of statins can reduce the risk of SNHL, but conflicting conclusions exist, and the relationship between statin use and hearing loss remains controversial.^[10,11]^ Furthermore, the impact of lipid-lowering drugs other than statins on SNHL is currently unclear, and conducting randomized controlled trials to investigate this issue is both time-consuming and expensive. Therefore, we aim to utilize Mendelian randomization (MR) methods to explore whether lipid levels and lipid-lowering drugs, including statins, are associated with SNHL.

MR is a powerful method that uses genetic variations as instrumental variables to infer causal relationships between exposures and outcomes.^[12]^ Within this framework, drug-target MR represents a specialized approach that exploits genetic variations within or near drug target genes to investigate the causal impact of drug target modulation on various health outcomes. This approach has been successfully employed in prior research to examine the effects of lipid-lowering drugs on conditions such as psoriasis and systemic lupus erythematosus,^[13,14]^ facilitating the repurposing of existing pharmaceuticals and yielding substantial healthcare cost savings. Given the pressing need for effective interventions in the realm of SNHL, we undertook a drug-target MR study to evaluate the potential effects of different lipid-lowering drugs on SNHL.

## 2. Methods

### 2.1. Study design and data sources

**Figure 1.**
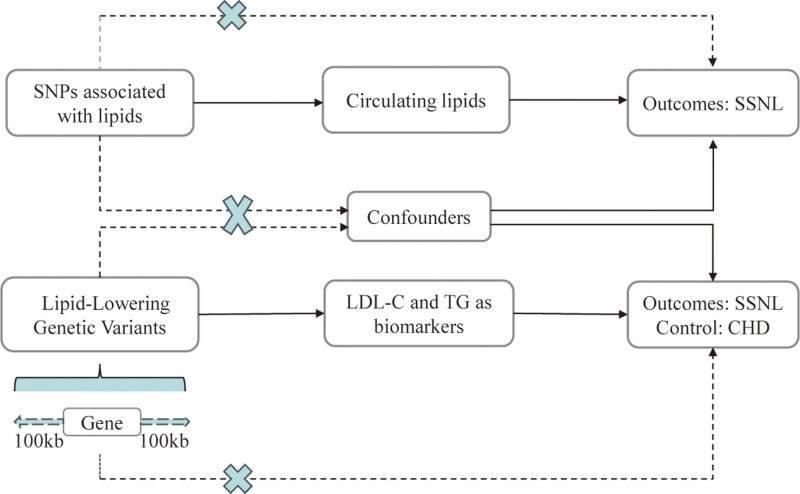
Flowchart on the overall study design.

The data utilized in this study were derived from publicly available, aggregated datasets obtained from genome-wide association studies (GWAS) and expression quantitative trait loci studies. The research design was divided into 2 distinct components, outlined in Fig. 1. In the initial phase, we employed a 2-sample univariable and multivariable MR approach to investigate the potential causal relationship between lipids and SNHL. This entailed analyzing lipid-related data sourced from a comprehensive GWAS study conducted by the esteemed UK Biobank. Specifically, we examined TG, LDL-C, high-density lipoprotein cholesterol (HDL-C), apolipoprotein A-I (APO A-1), and apolipoprotein B (APO B) as key lipid parameters. For the outcome analysis, we utilized SNHL GWAS data obtained from the Finnish database, accessible at https://www.finngen.fi/en/access_results. In the second phase, we undertook a drug-target MR analysis to explore the genetic associations between genes targeted by lipid-lowering drugs and SNHL. Classification of the lipid-lowering drugs and their respective target genes was based on pertinent studies and the most up-to-date guidelines governing lipid-lowering therapies.^[15,16]^ Comprehensive information regarding the relevant drug targets and their corresponding coding genes was sourced from DrugBank (https://go.drugbank.com/) and the NCBI Gene database (https://www.ncbi.nlm.nih.gov/gene/), respectively. Specific details are provided in Table 1, offering a succinct summary of the pertinent information. The data used in this study were sourced from public databases and had received ethical approval from the original studies. Therefore, no additional ethical approval was required for this study.

**Table 1:** Summary of information on lipid-lowering drug targets.

Drug effect	Drug class	Drug substance	Drug target	Target genes	Gene location (GRCh37 from Ensembl)	Instruments
*Reduced LDL-C*	Key modulater	–	LDL receptor	LDLR	CHR:19: 11200139–11244496	40
	HMGCR inhibitors	Atorvastatin, rosuvastatin, etc	HMG-CoA reductase	HMGCR	CHR:5: 74632993–74657941	12
	PCSK9 inhibitors	Evolocumab, alirocumab	Proprotein convertase subtilisin/kexin type 9	PCSK9	CHR:1: 55505221–55530525	27
	Cholesterol absorption inhibitors	Ezetimibe	Niemann-Pick C1-like 1	NPC1L1	CHR:7: 44552134–44580929	4
	ACLY inhibitors	Bempedoic acid	ATP-citrate synthase	ACLY	CHR:17: 40023170–40075275	0
	ASO targeting CETP mRNA	Torcetrapib	Cholesteryl ester transfer protein	CEPT	CHR:16: 56995862–57017757	8
	ASO targeting ApoB mRNA	Mipomersen	Apo B100	APOB	CHR:2: 21224301–21266945	20
*Reduced TG*	Key modulater	–	Lipoprotein lipase	LPL	CHR:8: 19796764–19824770	36
	Fibrates	Fenofibrate, gemfibrozil	Peroxisome proliferator-activated receptor-alpha	PPARA	CHR:22: 46546429–46639653	2
	ANGPTL3 inhibitors	Evinacumab	Angiopoietin-related protein 3	ANGPTL3	CHR:1: 63063191–63071984	11
	ASO targeting ApoC-III mRNA	Volanesorsen	Apo C-III	APOC3	CHR:11: 116700623–116703788	31

ACLY = ATP-citrate synthase, ANGPTL3 = angiopoietin-related protein 3, APO B = apolipoprotein B, APOC3 = apoprotein C-III, ASO = antisense oligonucleotide, CETP = cholesteryl ester transfer protein, HMG-CoA = 3-Hydroxy-3-methylglutaryl coenzyme A, HMGCR = 3-hydroxy-3-methylglutaryl-coenzyme A reductase, LDL-C = low-density lipoprotein cholesterol, LDLR = LDL receptor, LPL = lipoprotein lipase, NPC1L1= Niemann-Pick C1-like 1, PCSK9 = proprotein convertase subtilisin/kexin type 9, PPARA = peroxisome proliferator activated receptor alpha, TG = triglycerides.

### 2.2. Selection of genetic variants

In the 2-sample MR study, single nucleotide polymorphisms (SNPs) associated with circulating lipid levels (TG, LDL-C, HDL-C, APO A-1, and APO B) were selected (*P* < 5e−8), and they met the linkage disequilibrium (LD) clumping threshold of *r*^2^ < 0.001 and a physical distance threshold of 10,000 kb. In the drug-target MR study, a total of 11 target genes were identified. Based on their primary pharmacological effects, LDL receptor (LDLR), HMGCR, proprotein convertase subtilisin/kexin type 9, ATP-citrate synthase, Niemann-Pick C1-like 1 (NPC1L1), cholesteryl ester transfer protein (CETP), and APO B-100 were selected as target genes for lowering LDL-C levels; while lipoprotein lipase, angiopoietin-related protein 3 (ANGPTL3), apoprotein C-III (APOC3), and peroxisome proliferator activated receptor alpha (PPARA) were selected as target genes for lowering TG levels. According to the different target genes controlled by the above genes, we extracted the relevant SNPs from the GWAS data for LDL-C and TG aggregated from the UK Biobank. The specific approach involved selecting SNPs within ±100 kb of the respective target genes and setting LD threshold *r*^2^ < 0.2 and a physical distance threshold of 250 kb. As no SNPs related to ATP-citrate synthase were extracted, it was excluded from the subsequent analysis. Ultimately, our analysis included 10 target genes: LDLR, HMGCR, PCKS9, NPC1L1, CETP, APOB, LPL, ANGPTL3, PPARA, and APOC3.

### 2.3. Statistical analysis

In this study, inverse variance weighted method^[17]^ was used to estimate the causal effects of genetic proxies for circulating lipids and lipid-lowering treatment targets on SNHL. This approach estimates the causal relationship associated with a one standard deviation increase in exposure to the genetically predicted factor for the outcome. To test the MR assumptions, we first calculated the *F* statistic for each instrumental variable using the formula *F* = *R*^2^/(1 − *R*^2^) * (n − *k* − 1)/*k*, where *R*^2^ = 2(1 − minor allele frequency) * minor allele frequency * β^2^, with *R*^2^ representing the proportion of variance in the trait explained by genetic variation, *k* indicating the count of valid instrumental variables, and n representing the sample size. SNPs with *F* statistics >10 were selected to avoid weak instrument bias.^[18]^ Subsequently, sensitivity tests using MR-Egger regression and weighted median method were performed to validate the results from the inverse variance weighted method. Cochran *Q* test and MR Egger intercept test were conducted for heterogeneity and horizontal pleiotropy testing. The “leave-one-out” method was employed to identify heterogeneous SNPs. To validate the validity of the selected drug target gene variants, a positive control analysis was conducted using coronary heart disease (CHD) as the outcome, with GWAS summary data for CHD obtained from the Coronary Artery Disease Genetics Consortium (CARDIoGRAMplusC4D). Bonferroni correction was further applied to adjust for multiple testing, with a significance threshold of *P* < .01 (0.05/5) for the correction of the 5 lipid traits and *P* < .005 (.05/10) for the analysis of the 10 drug target genes. Uncorrected *P* values < .05 were considered suggestive associations, while Bonferroni-corrected *P* values reaching the significance threshold indicated statistically significant evidence for the significant relationship between the target gene and SNHL risk.^[19]^

## 3. Result

### 3.1. The impact of circulating lipid levels on SNHL

The instrumental variable information for the 5 lipid traits (TG, LDL-C, HDL-C, APO A-1, and APO B) can be found in Tables S3–S7, Supplemental Digital Content, https://links.lww.com/MD/P779, and all instrumental variables have *F* statistics exceeding 10.

**Figure 2.**
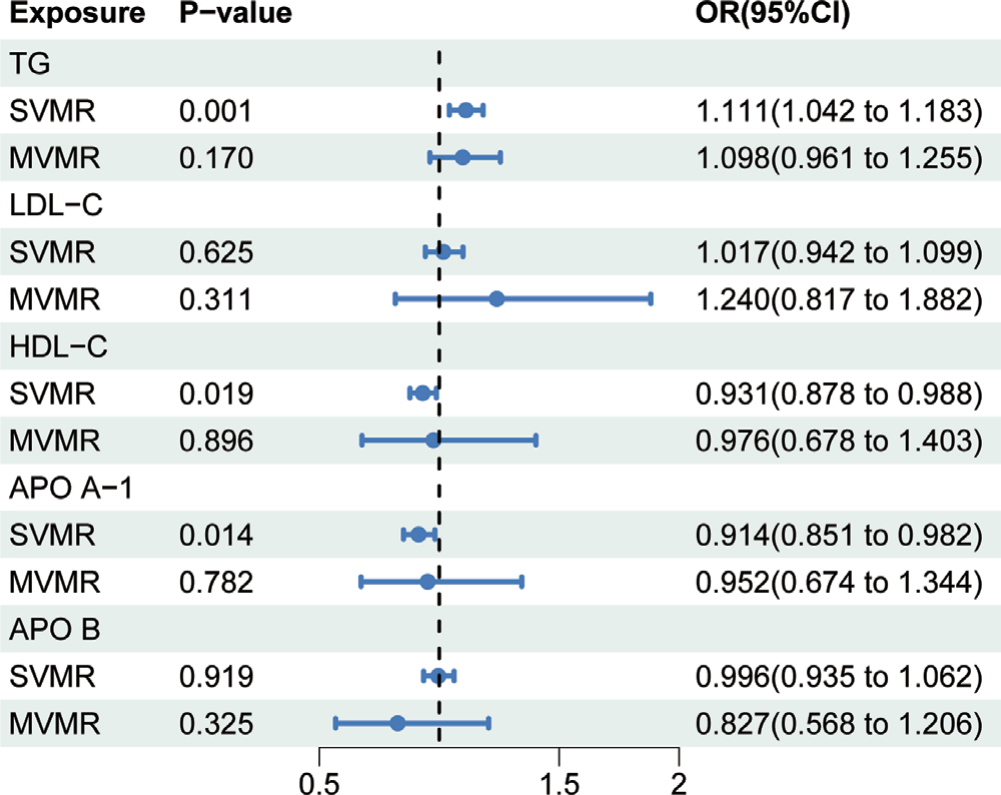
Single-variable and multivariate Mendelian randomization of lipid profiles and hearing loss. APO A-1 = apolipoprotein A-I, APO B = apolipoprotein B, HDL-C = high-density lipoprotein cholesterol, LDL-C = low-density lipoprotein cholesterol, MVMR = multivariate Mendelian randomization, SVMR = single-variable Mendelian randomization, TG = triglycerides.

In the univariate MR analysis, genetically predicted TG (OR = 1.111 [95% CI, 1.042–1.183]; *P* = .001) was associated with an increased risk of SNHL (Fig. 2, Table S1, Supplemental Digital Content, https://links.lww.com/MD/P780), and the estimate reached statistical significance after Bonferroni correction. HDL-C (OR = 0.931 [95% CI, 0.878–0.988]; *P* = .019) and APO A-1 (OR = 0.914 [95% CI, 0.851–0.982]; *P* = .014) were associated with a decreased risk of SNHL (Fig. 2, Table S1, Supplemental Digital Content, https://links.lww.com/MD/P780), but did not pass Bonferroni correction. In the multivariable MR analysis adjusting for the lipid traits, no significant associations with SNHL were observed (Fig. 2, Table S1, Supplemental Digital Content, https://links.lww.com/MD/P780). Sensitivity analyses demonstrated consistent trends in the estimates, and Cochran *Q* test indicated heterogeneity for TG, LDL-C, and APO A-1, although no horizontal pleiotropy was detected for these 5 lipid traits through MR-Egger analysis (Table S1, Supplemental Digital Content, https://links.lww.com/MD/P780). Omission of one sensitivity analysis showed stable results (Figs. S1–S5, Supplemental Digital Content, https://links.lww.com/MD/P781).

### 3.2. Lipid‑lowering drug targets and SNHL

**Figure 3.**
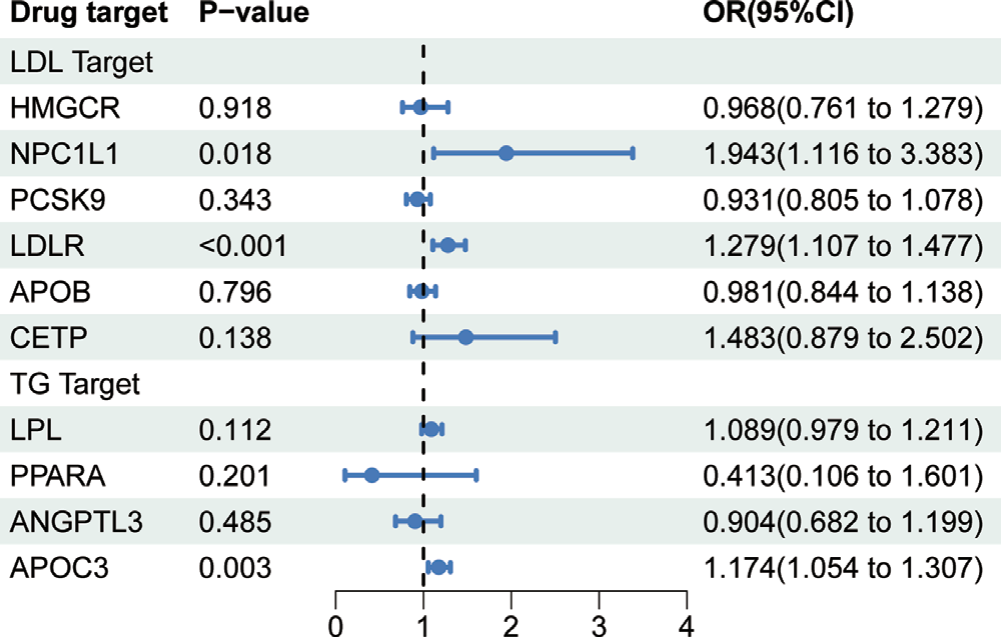
Mendelian randomization results for lipid-lowering drug targets and hearing loss. ANGPTL3 = angiopoietin-related protein 3, APO B = apolipoprotein B, APOC3 = apoprotein C-III, CETP = cholesteryl ester transfer protein, HMGCR = 3-hydroxy-3-methylglutaryl-coenzyme A reductase, LDLR = LDL receptor, LPL = lipoprotein lipase, NPC1L1= Niemann-Pick C1-like 1, PCSK9 = proprotein convertase subtilisin/kexin type 9, PPARA = peroxisome proliferator activated receptor alpha, TG = triglycerides.

The instrumental variable information for the 10 lipid-lowering drug target genes (HMGCR, PCSK9, NPC1L1, LDLR, APOB, CETP, LPL, ANGPTL3, APOC3, and PPARA) is provided in Tables S8–S17, Supplemental Digital Content, https://links.lww.com/MD/P779, with all instrumental variables having *F*-statistics exceeding 10, indicating the absence of weak instrument bias. In our positive control analysis for CHD, we observed significant associations between genetic proxy drug targets and reduced risk of CHD for all genes except ANGPTL3 (Fig. 3), demonstrating the effectiveness of the selected genetic instruments.

As shown in Fig. 2, the genetically predicted variants associated with LDL-C reduction in NPC1L1 (OR = 1.943 [95% CI, 1.116–3.383]; *P* = .018), LDLR (OR = 1.279 [95% CI, 1.107–1.477]; *P* < .001), and TG reduction in APOC3 (OR = 1.174 [95% CI, 1.054–1.307]; *P* = .003) were associated with an increased risk of SNHL. After Bonferroni correction, the associations of LDLR and APOC3 variants with increased SNHL risk remained significant, while the association with NPC1L1 variant became nonsignificant. The remaining target genes showed a protective trend in regulating SNHL risk, except for the lipoprotein lipase lipid-lowering variant, which exhibited a tendency towards risk, although none reached statistical significance (Fig. 2). Sensitivity analyses did not reveal evidence of heterogeneity or horizontal pleiotropy (Table S2, Supplemental Digital Content, https://links.lww.com/MD/P780). Omission of one sensitivity analysis confirmed the robustness of the results (Figs. S6–S14, Supplemental Digital Content, https://links.lww.com/MD/P781).

## 4. Discussion

In this study, there is no genetic evidence to suggest a causal relationship between circulating lipid levels and SNHL. However, genetic proxies simulating the lowering of LDL-C through variants in LDLR and the lowering of TG through variants in APOC3 are associated with an increased risk of SNHL. The observed risk increase appears to be unrelated to LDL-C or TG control, as there is no clear evidence that LDL-C and TG have an impact on the risk of SNHL. This provides new insights into the prevention, treatment, and other applications of lipid-lowering drugs for SNHL.

Statins, as inhibitors of 3-Hydroxy-3-methylglutaryl coenzyme A reductase, are commonly used lipid-lowering drugs. They achieve their cholesterol-lowering effects by competitively inhibiting 3-Hydroxy-3-methylglutaryl coenzyme A reductase, which leads to the suppression of cholesterol synthesis. Statins exert a protective role in cardiovascular diseases by reducing the activity of the cholesterol synthesis pathway.^[20]^ Additionally, they also play a role in reducing oxidative stress, inflammation, and immune regulation.^[21]^ The pleiotropic effects of statins have expanded their clinical applications, such as in neurological disorders like Parkinson and dementia.^[22,23]^ Previous experiments and clinical studies^[11,24]^ have reported on the impact of statins on hearing loss, but their relationship appears to be controversial. Some studies have suggested that participants using statins had a 48% lower risk of hearing impairment compared to nonusers.^[25]^ The lipid-lowering and atherosclerosis-reducing effects of statins may contribute to their protective effect on the ears.^[26]^ On the other hand, statins may increase the risk of sudden SNHL. Research has indicated a positive correlation between prior statin use in individuals with normal blood lipids and sudden SNHL .^[27]^ Furthermore, a case report described irreversible mid-frequency SNHL after 18 months of atorvastatin treatment,^[28]^ possibly related to harmful immune modulation of cochlear hair cells or vestibular dysfunction caused by decreased cochlear cholesterol. Although our study found a protective trend of HMGCR inhibition on hearing, the results did not reach significance.

The LDLR participates in endocytosis, promoting the transport and clearance of LDL-C from the plasma to the cytoplasm. This process primarily occurs in the liver, removing approximately 70% of low-density lipoproteins from circulation and thus affecting the levels of LDL in the plasma.^[29,30]^ The LDLR gene is located on the short arm of human chromosome 19 and consists of 18 exons and 17 introns, with a total length of 45 kb. Previous studies have found that LDLR gene polymorphisms are closely related to lipid metabolism, and lipid metabolism has a strong correlation with the formation of early atherosclerotic plaques.^[31]^ Microcirculatory disorders are considered one of the mechanisms underlying sudden SNHL, and lipid metabolism is related to hemorheology. The presence of lipids adhering to the surface of red blood cells and platelets has been shown to have a detrimental effect on the charge-carrying capacity of red blood cells, increasing the viscosity between cells and ultimately leading to a decrease in blood flow.^[32]^ This can result in a reduction in the blood supply to the inner ear, ultimately leading to SNHL. Such lipid accumulation can also have a direct impact on platelet function and aggregation, further exacerbating the adverse effects on blood flow and contributing to the development of SNHL. Therefore, in theory, the expression of LDLR could be a protective factor for SNHL by reducing LDL-C in circulation. Recent studies have indicated a higher susceptibility to sudden deafness associated with LDLR gene polymorphisms, but no association with blood lipid levels has been found. This may be due to insufficient sample size and sample imbalance. We found that LDLR variants associated with lipid lowering increase the risk of SNHL, but the relationship between serum LDL-C and SNHL is not significant. LDLR may affect SNHL through other pathways rather than LDL-C. Studies have shown that LDLR is highly expressed in cochlear cells involved in lymphatic homeostasis, and after the occurrence of hearing loss, LDLR is also expressed in non-sensory supporting cells that regulate neighboring outer hair cell electromotility and cochlear amplification.^[30]^ Further research is needed to determine whether LDLR is associated with SNHL. Regarding APOC3, increasing evidence suggests that elevated plasma levels of ApoC3 are positively correlated with increased circulating triglyceride concentration and cardiovascular disease (CVD) incidence.^[33]^ Human knockout studies of ApoC3 have shown that ApoC3 deficiency lowers plasma triglyceride and cholesterol levels and increases HDL-C levels.^[34]^ From a microcirculatory perspective, the inhibition of APOC3 is a protective factor for SNHL. However, recent MR studies have found that only one rare loss-of-function variant of APOC3 (rs138326449) in the European population is associated with decreased plasma triglyceride levels and reduced CVD risk, while several common loss-of-function variants do not exhibit cardiac protective characteristics.^[35]^ In addition, the absence of ApoC3 in LDLR^−/−^ mice has no effect on lipid metabolism and the formation of atherosclerosis,^[36]^ although there are differences between animal and human experiments, at least proving that the role of ApoC3 inhibition in hyperlipidemia and cardiovascular disease still needs further verification.

When interpreting the results of this study, certain limitations should be taken into consideration. Firstly, the genetic variants used in this study were designed to simulate the impact of decreased lipid levels on the risk of SNHL, and may not directly translate to the effects of lipid-lowering drugs. Nonetheless, these findings still provide valuable insights into SNHL, but further clinical research is required to validate these results. Secondly, although sensitivity analysis demonstrated the stability of the research findings, the existence of pleiotropy cannot be completely ruled out, whereby genetic variations influence exposure and outcomes through different pathways. Lastly, due to the limitations of GWAS data, our study results are specific to the European population, as gene associations and environmental factors may vary across populations, thus restricting the generalizability of the findings to other populations. To verify the generalizability of our research results, further studies involving diverse populations are necessary.

## 5. Conclusion

This study provides genetic evidence demonstrating a potential causal impact of lipid-lowering drugs on increased risk of SNHL through the LDLR and APOC3 pathways. However, further research is needed to better understand the underlying mechanisms and the results should be evaluated through clinical trials.

## Author contributions

**Conceptualization:** Kun-Lin Pu.

**Data curation:** Yan Yang, Hao-Fei Huang.

**Formal analysis:** Hao-Fei Huang.

**Validation:** Hao-Fei Huang.

**Writing – original draft:** Yan Yang, Kun-Lin Pu.

## Supplementary Material


