# Supplementary material for: Exploring the relationship between lipid-lowering drug target genes and sensorineural hearing loss by Mendelian randomization
Source: Medicine (Baltimore). 2025 Aug 29;104(35):e44174. doi: 10.1097/MD.0000000000044174 (PMC12401366; doi:10.1097/MD.0000000000044174)
Supplement: Supplementary file 2 [file medi-104-e44174-s002.pdf]

Supplementary Table 1: Results of MR and sensitivity analyses of lipids with SNHL

Exposure	Method	NSNP	OR(95%CI)	P-value	Q-pval	Intercept P-value
**TG**						
SVMR	MR Egger	284	1.094(0.996-1.202)	0.061	-	0.668
	WM	284	1.057(0.958-1.166)	0.266	-	-
	IVW	284	1.111(1.042-1.183)	0.001	0.038	-
MVMR	IVW	180	1.098(0.961-1.255)	0.170	-	-
**LDL-C**						
SVMR	MR Egger	158	0.993(0.886-1.112)	0.905	-	0.561
	WM	158	1.034(0.921-1.162)	0.570	-	-
	IVW	158	1.017(0.942-1.099)	0.625	0.023	-
MVMR	IVW	111	1.240(0.817-1.882)	0.311	-	-
**HDL-C**						
SVMR	MR Egger	326	0.942(0.861-1.031)	0.198	-	0.741
	WM	326	0.975(0.882-1.078)	0.633	-	-
	IVW	326	0.931(0.878-0.988)	0.019	0.171	-
MVMR	IVW	253	0.976(0.678-1.403)	0.896	-	-
**APO A-1**						
SVMR	MR Egger	269	0.962(0.857-1.081)	0.521	-	0.269
	WM	269	0.985(0.886-1.095)	0.791	-	-
	IVW	269	0.914(0.851-0.982)	0.014	<0.001	-
MVMR	IVW	217	0.952(0.674-1.344)	0.782	-	-
**APO B**						
SVMR	MR Egger	184	0.965(0.886-1.052)	0.423	-	0.280
	WM	184	0.971(0.874-1.077)	0.577	-	-
	IVW	184	0.996(0.935-1.062)	0.919	0.099	-
MVMR	IVW	128	0.827(0.568-1.206)	0.325	-	-

Supplementary Table 2: Results of MR and sensitivity analyses of lipid-lowering drug targets with SNHL

Drug target	Outcome	NSNP	OR(95%CI)	P-value	Q-pval	Intercept P-value
**LDL Target**						
HMGCR	SSNL	12	0.986 (0.761-1.279)	0.918	0.565	0.152
	CHD	12	0.583 (0.477-0.714)	<0.001	0.867	0.465
NPC1L1	SSNL	4	1.943 (1.116-3.383)	0.018	0.893	0.826
	CHD	4	0.496(0.304-0.809)	0.004	0.931	0.607
PCSK9	SSNL	27	0.931 (0.805-1.078)	0.343	0.864	0.169
	CHD	24	0.451 (0.378-0.537)	<0.001	0.576	0.466
LDLR	SSNL	40	1.279 (1.107-1.477)	0.0008	0.530	0.552
	CHD	34	0.412 (0.352-0.483)	<0.001	0.001	0.148
APOB	SSNL	20	0.981(0.844-1.138)	0.796	0.512	0.523
	CHD	19	0.707(0.578-0.866)	<0.001	<0.001	0.136
CETP	SSNL	8	1.483(0.879 -2.502)	0.138	0.408	0.101
	CHD	8	0.477(0.325-0.699)	<0.001	0.817	0.954
**TG Target**						
LPL	SSNL	36	1.089(0.979-1.211)	0.112	0.313	0.691
	CHD	32	0.676(0.622-0.734)	<0.001	0.885	0.202
PPARA	SSNL	2	0.413(0.106-1.601)	0.201	0.856	-
	CHD	2	0.247(0.091-0.666)	0.005	0.710	-
ANGPTL3	SSNL	11	0.904 (0.682-1.199)	0.485	0.384	0.225
	CHD	10	0.853(0.698-1.041)	0.118	0.949	0.984
APOC3	SSNL	31	1.174(1.054-1.307)	0.003	0.085	0.361
	CHD	31	0.795(0.743-0.851)	<0.001	0.719	0.174
