# Supplementary material for: Exploring the relationship between lipid-lowering drug target genes and sensorineural hearing loss by Mendelian randomization
Source: Medicine (Baltimore). 2025 Aug 29;104(35):e44174. doi: 10.1097/MD.0000000000044174 (PMC12401366; doi:10.1097/MD.0000000000044174)
Supplement: Supplementary file 3 [file medi-104-e44174-s003.pdf]

Supplementary Figures1: MR leave−one−out sensitivity analysis for TG.

Supplementary Figures2: MR leave−one−out sensitivity analysis for LDL.

Supplementary Figures3: MR leave−one−out sensitivity analysis for HDL.

Supplementary Figures4: MR leave−one−out sensitivity analysis for APO A-1.

Supplementary Figures5: MR leave−one−out sensitivity analysis for APO B.

Supplementary Figures6: MR leave−one−out sensitivity analysis for HMGCR.

Supplementary Figures7: MR leave−one−out sensitivity analysis for NPC1L1.

Supplementary Figures8: MR leave−one−out sensitivity analysis for PCSK9.

Supplementary Figures9: MR leave−one−out sensitivity analysis for APOB.

Supplementary Figures10: MR leave−one−out sensitivity analysis for CETP.

Supplementary Figures11: MR leave−one−out sensitivity analysis for LDLR.

Supplementary Figures12: MR leave−one−out sensitivity analysis for LPL.

Supplementary Figures13: MR leave−one−out sensitivity analysis for ANGPTL3.

Supplementary Figures14: MR leave−one−out sensitivity analysis for APOC3.

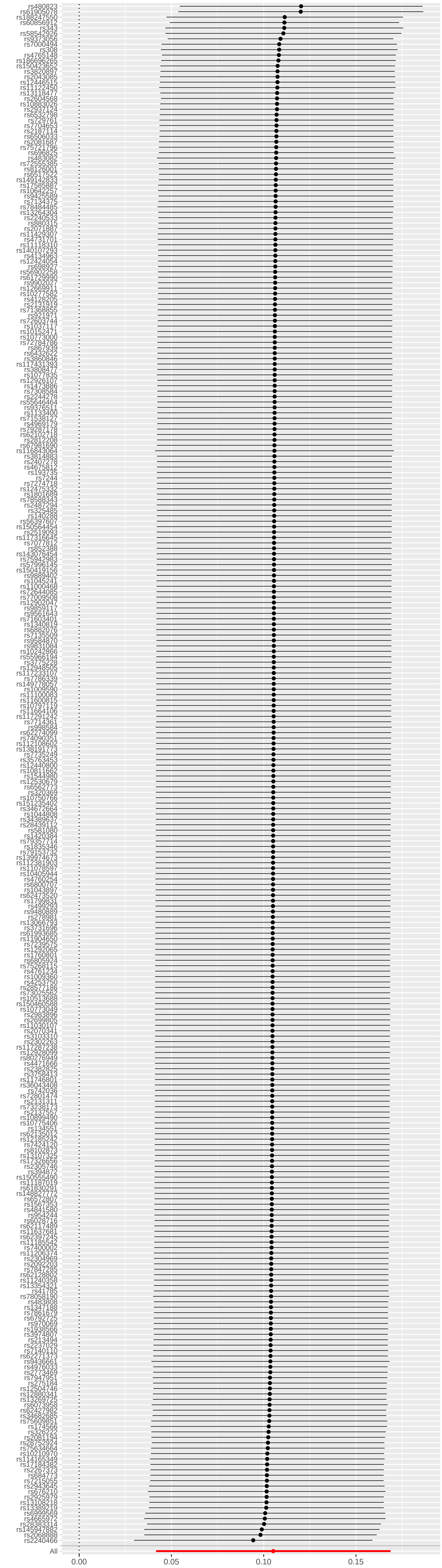

MR leave−one−out sensitivity analysis for
'triglycerides || id:ieu−b−111' on 'Sensorineural hearing loss || id:finn−b−H8_HL_SEN_NAS'

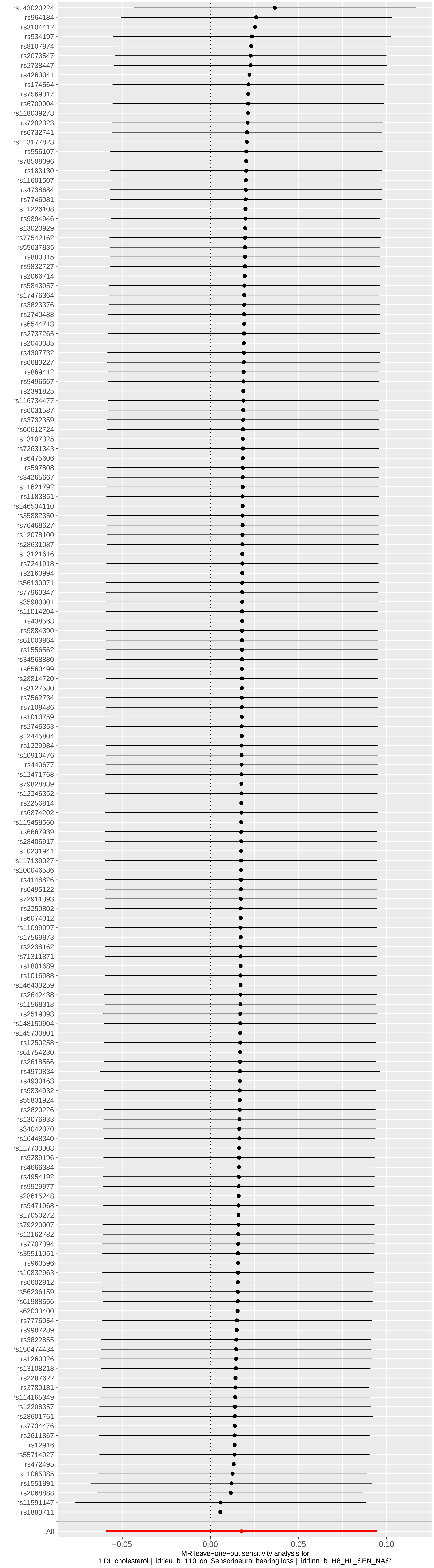
rs143020224
rs964184
rs3104412
rs934197
rs8107974
rs2073547
rs2738447
rs4263041
rs174564
rs7569317
rs6709904
rs118039278
rs7202323
rs6732741
rs113177823
rs556107
rs78508096
rs183130
rs11601507
rs4738684
rs7746081
rs11226108
rs9894946
rs13020929
rs77542162
rs55637835
rs880315
rs9832727
rs2066714
rs5843957
rs17476364
rs3823376
rs2740488
rs6544713
rs2737265
rs2043085
rs4307732
rs6680227
rs869412
rs9496567
rs2391825
rs116734477
rs6031587
rs3732359
rs60612724
rs13107325
rs72631343
rs6475606
rs597808
rs34265667
rs11621792
rs1183851
rs146534110
rs35882350
rs76468627
rs12078100
rs28631087
rs13121616
rs7241918
rs2160994
rs56130071
rs77960347
rs35980001
rs11014204
rs438568
rs9884390
rs61003864
rs1556562
rs34568880
rs6560499
rs28814720
rs3127580
rs7562734
rs7108486
rs1010759
rs2745353
rs12445804
rs1229984
rs10910476
rs440677
rs12471768
rs79828839
rs12246352
rs2256814
rs6874202
rs115458560
rs6667939
rs28406917
rs10231941
rs117139027
rs200046586
rs4148826
rs6495122
rs72911393
rs2250802
rs6074012
rs11099097
rs17569873
rs2238162
rs71311871
rs1801689
rs1016988
rs146433259
rs2642438
rs11568318
rs2519093
rs148150904
rs145730801
rs1250258
rs61754230
rs2618566
rs4970834
rs4930163
rs9834932
rs55831924
rs2820226
rs13076933
rs34042070
rs10448340
rs117733303
rs9289196
rs4666384
rs4954192
rs9929977
rs28615248
rs9471968
rs17050272
rs79220007
rs12162782
rs7707394
rs35511051
rs960596
rs10832963
rs6602912
rs56236159
rs61988556
rs62033400
rs7776054
rs9987289
rs3822855
rs150474434
rs1260326
rs13108218
rs2287622
rs3780181
rs114165349
rs12208357
rs28601761
rs7734476
rs2611867
rs12916
rs55714927
rs472495
rs11065385
rs1551891
rs2068888
rs11591147
rs1883711
All
−0.05
0.00
0.05
0.10
MR leave−one−out sensitivity analysis for
'LDL cholesterol || id:ieu−b−110' on 'Sensorineural hearing loss || id:finn−b−H8_HL_SEN_NAS'

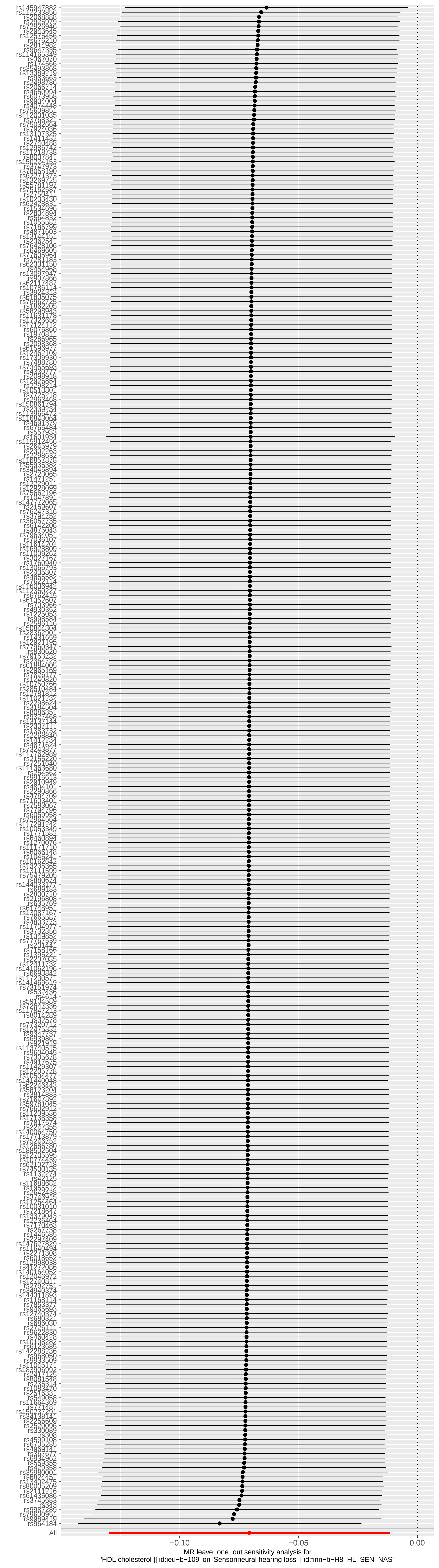
All
−0.10
−0.05
0.00
MR leave−one−out sensitivity analysis for
'HDL cholesterol || id:ieu−b−109' on 'Sensorineural hearing loss || id:finn−b−H8_HL_SEN_NAS'

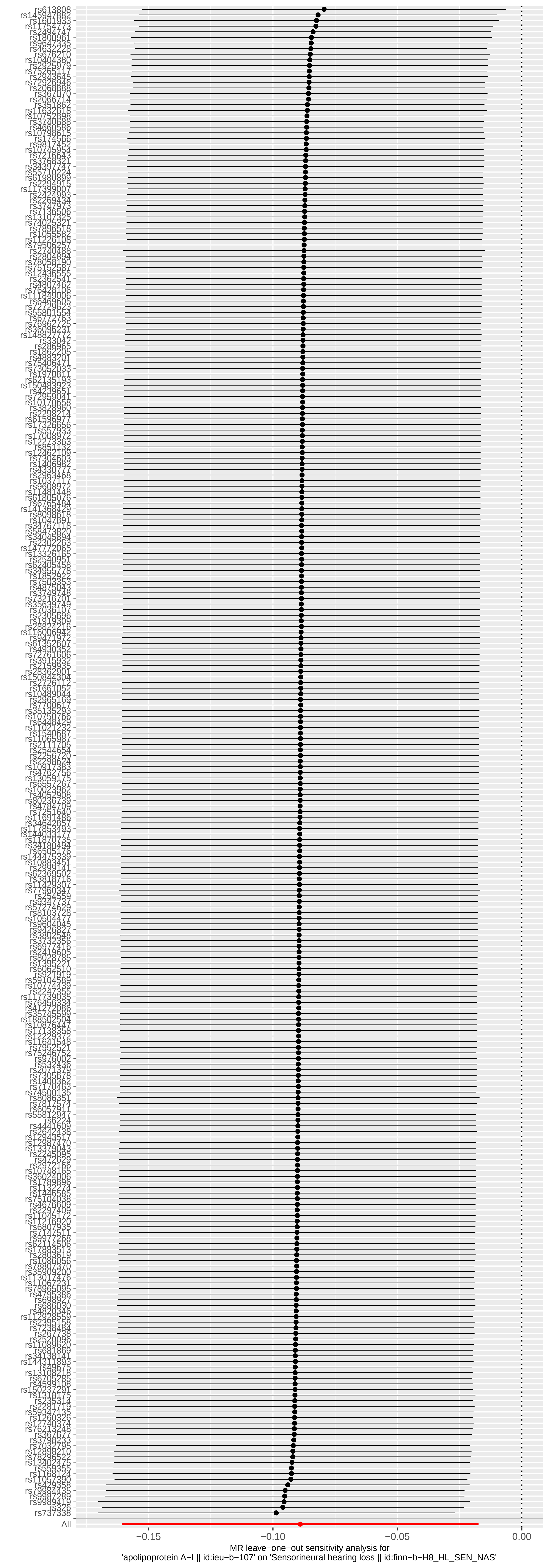
rs613808
rs737338
All
−0.15
−0.10
−0.05
0.00
MR leave−one−out sensitivity analysis for
'apolipoprotein A−I || id:ieu−b−107' on 'Sensorineural hearing loss || id:finn−b−H8_HL_SEN_NAS'

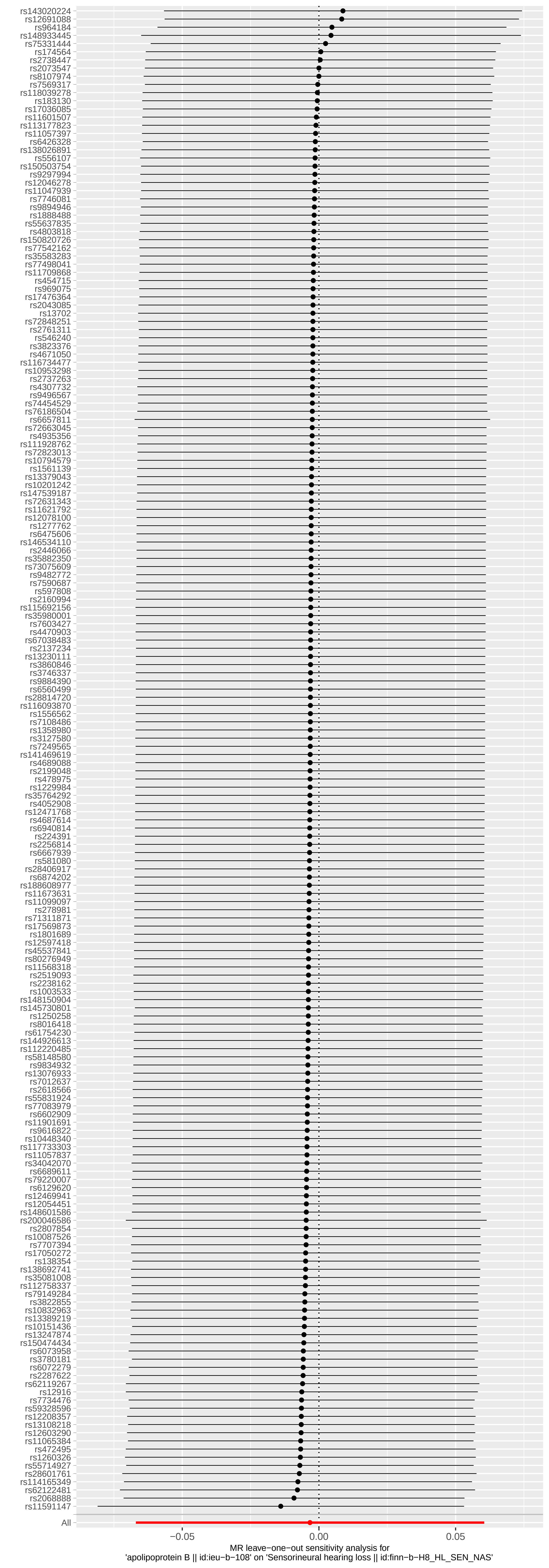
rs143020224
rs12691088
rs964184
rs148933445
rs75331444
rs174564
rs2738447
rs2073547
rs8107974
rs7569317
rs118039278
rs183130
rs17036085
rs11601507
rs113177823
rs11057397
rs6426328
rs138026891
rs556107
rs150503754
rs9297994
rs12046278
rs11047939
rs7746081
rs9894946
rs1888488
rs55637835
rs4803818
rs150820726
rs77542162
rs35583283
rs77498041
rs11709868
rs454715
rs969075
rs17476364
rs2043085
rs13702
rs72848251
rs2761311
rs546240
rs3823376
rs4671050
rs116734477
rs10953298
rs2737263
rs4307732
rs9496567
rs74454529
rs76186504
rs6657811
rs72663045
rs4935356
rs111928762
rs72823013
rs10794579
rs1561139
rs13379043
rs10201242
rs147539187
rs72631343
rs11621792
rs12078100
rs1277762
rs6475606
rs146534110
rs2446066
rs35882350
rs73075609
rs9482772
rs7590687
rs597808
rs2160994
rs115692156
rs35980001
rs7603427
rs4470903
rs67038483
rs2137234
rs13230111
rs3860846
rs3746337
rs9884390
rs6560499
rs28814720
rs116093870
rs1556562
rs7108486
rs1358980
rs3127580
rs7249565
rs141469619
rs4689088
rs2199048
rs478975
rs1229984
rs35764292
rs4052908
rs12471768
rs4687614
rs6940814
rs224391
rs2256814
rs6667939
rs581080
rs28406917
rs6874202
rs188608977
rs11673631
rs11099097
rs278981
rs71311871
rs17569873
rs1801689
rs12597418
rs45537841
rs80276949
rs11568318
rs2519093
rs2238162
rs1003533
rs148150904
rs145730801
rs1250258
rs8016418
rs61754230
rs144926613
rs112220485
rs58148580
rs9834932
rs13076933
rs7012637
rs2618566
rs55831924
rs77083979
rs6602909
rs11901691
rs9616822
rs10448340
rs117733303
rs11057837
rs34042070
rs6689611
rs79220007
rs6129620
rs12469941
rs12054451
rs148601586
rs200046586
rs2807854
rs10087526
rs7707394
rs17050272
rs138354
rs138692741
rs35081008
rs112758337
rs79149284
rs3822855
rs10832963
rs13389219
rs10151436
rs13247874
rs150474434
rs6073958
rs3780181
rs6072279
rs2287622
rs62119267
rs12916
rs7734476
rs59328596
rs12208357
rs13108218
rs12603290
rs11065384
rs472495
rs1260326
rs55714927
rs28601761
rs114165349
rs62122481
rs2068888
rs11591147
All
−0.05
0.00
0.05
MR leave−one−out sensitivity analysis for
'apolipoprotein B || id:ieu−b−108' on 'Sensorineural hearing loss || id:finn−b−H8_HL_SEN_NAS'

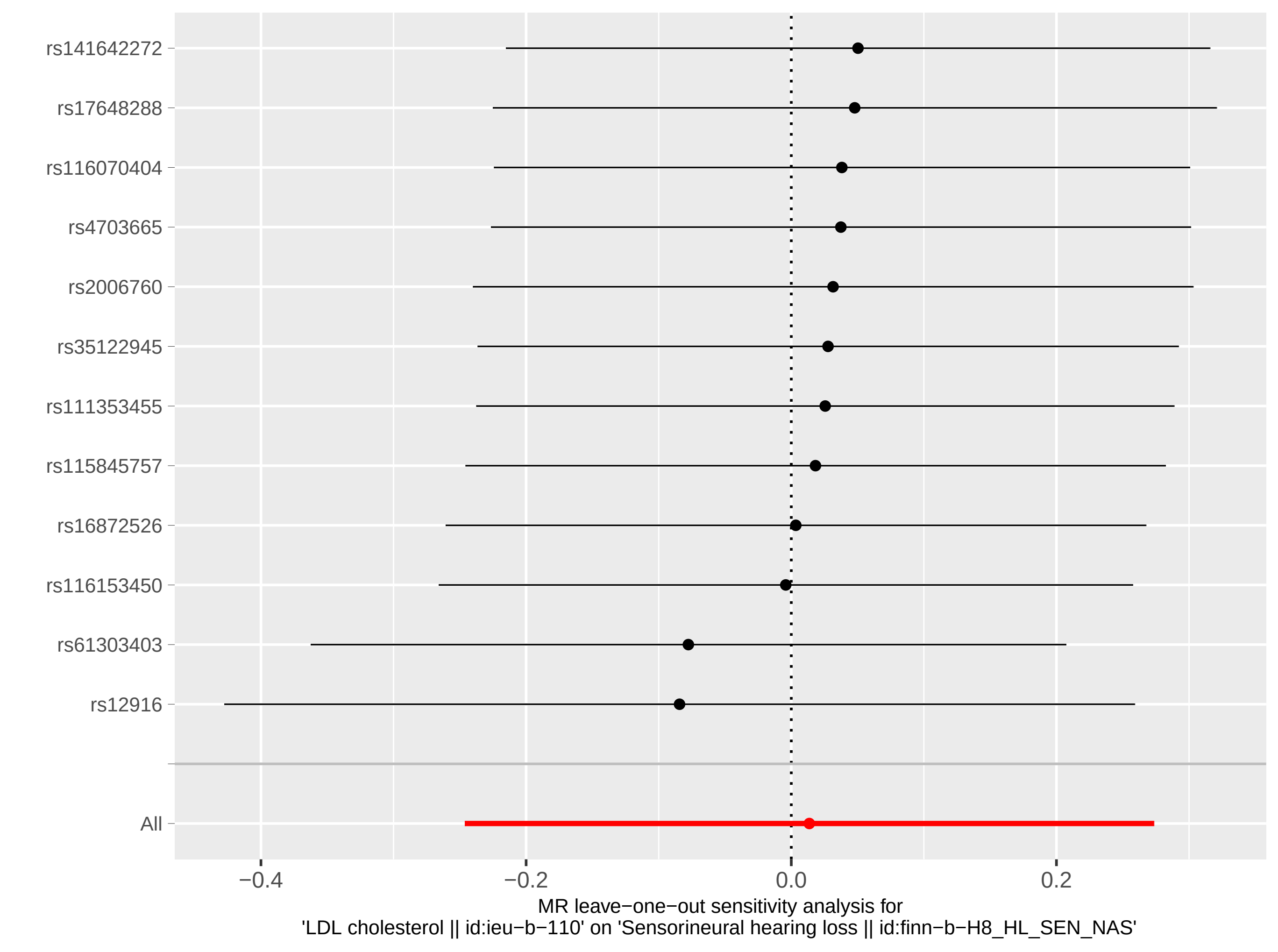
rs141642272
rs17648288
rs116070404
rs4703665
rs2006760
rs35122945
rs111353455
rs115845757
rs16872526
rs116153450
rs61303403
rs12916
All
−0.4
−0.2
0.0
0.2
MR leave−one−out sensitivity analysis for
'LDL cholesterol || id:ieu−b−110' on 'Sensorineural hearing loss || id:finn−b−H8_HL_SEN_NAS'

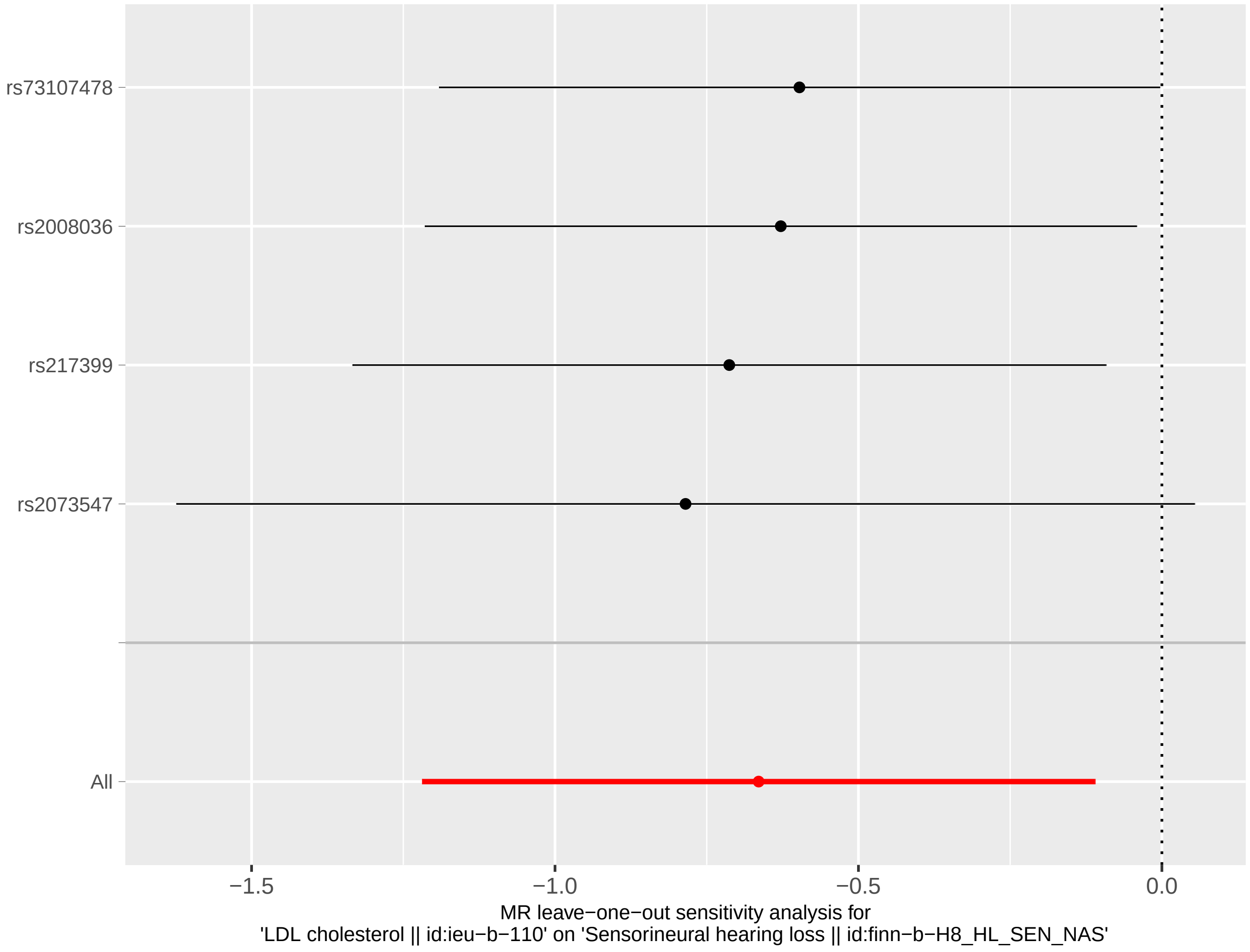
rs73107478
rs2008036
rs217399
rs2073547
All
−1.5
−1.0
−0.5
0.0
MR leave−one−out sensitivity analysis for
'LDL cholesterol || id:ieu−b−110' on 'Sensorineural hearing loss || id:finn−b−H8_HL_SEN_NAS'

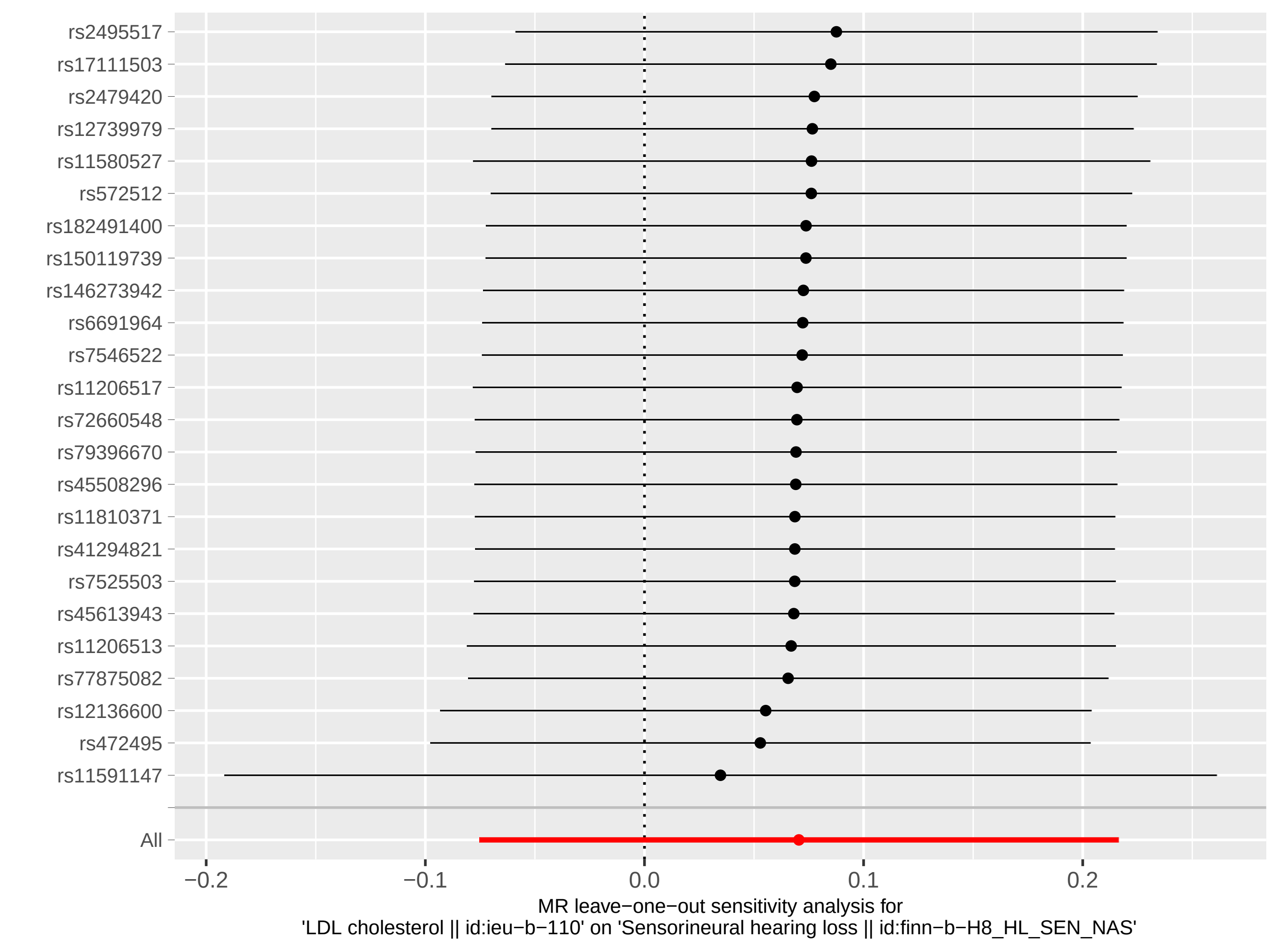
rs2495517
rs17111503
rs2479420
rs12739979
rs11580527
rs572512
rs182491400
rs150119739
rs146273942
rs6691964
rs7546522
rs11206517
rs72660548
rs79396670
rs45508296
rs11810371
rs41294821
rs7525503
rs45613943
rs11206513
rs77875082
rs12136600
rs472495
rs11591147
All
−0.2
−0.1
0.0
0.1
0.2
MR leave−one−out sensitivity analysis for
'LDL cholesterol || id:ieu−b−110' on 'Sensorineural hearing loss || id:finn−b−H8_HL_SEN_NAS'

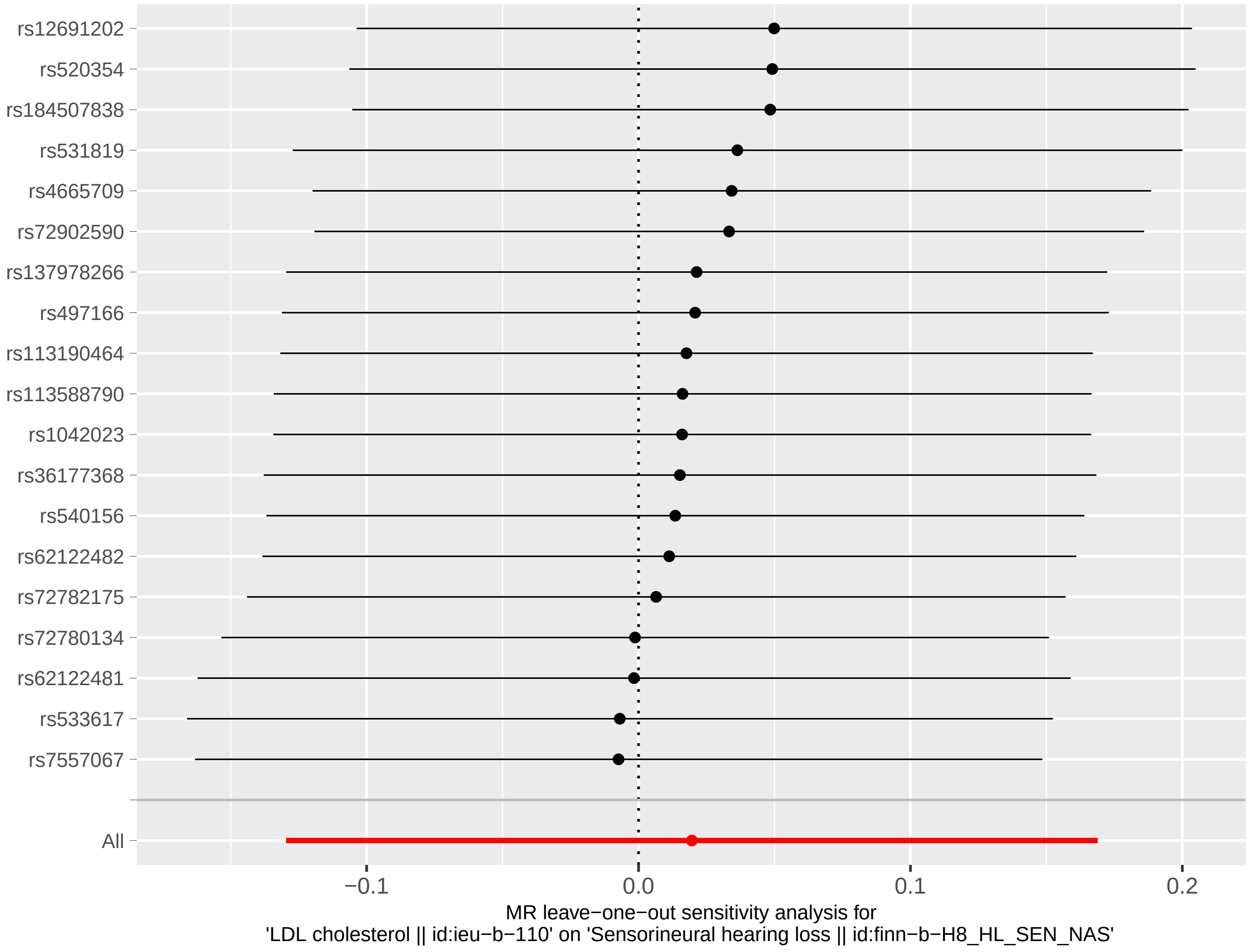
rs12691202
rs520354
rs184507838
rs531819
rs4665709
rs72902590
rs137978266
rs497166
rs113190464
rs113588790
rs1042023
rs36177368
rs540156
rs62122482
rs72782175
rs72780134
rs62122481
rs533617
rs7557067
All
−0.1
0.0
0.1
0.2
MR leave−one−out sensitivity analysis for
'LDL cholesterol || id:ieu−b−110' on 'Sensorineural hearing loss || id:finn−b−H8_HL_SEN_NAS'

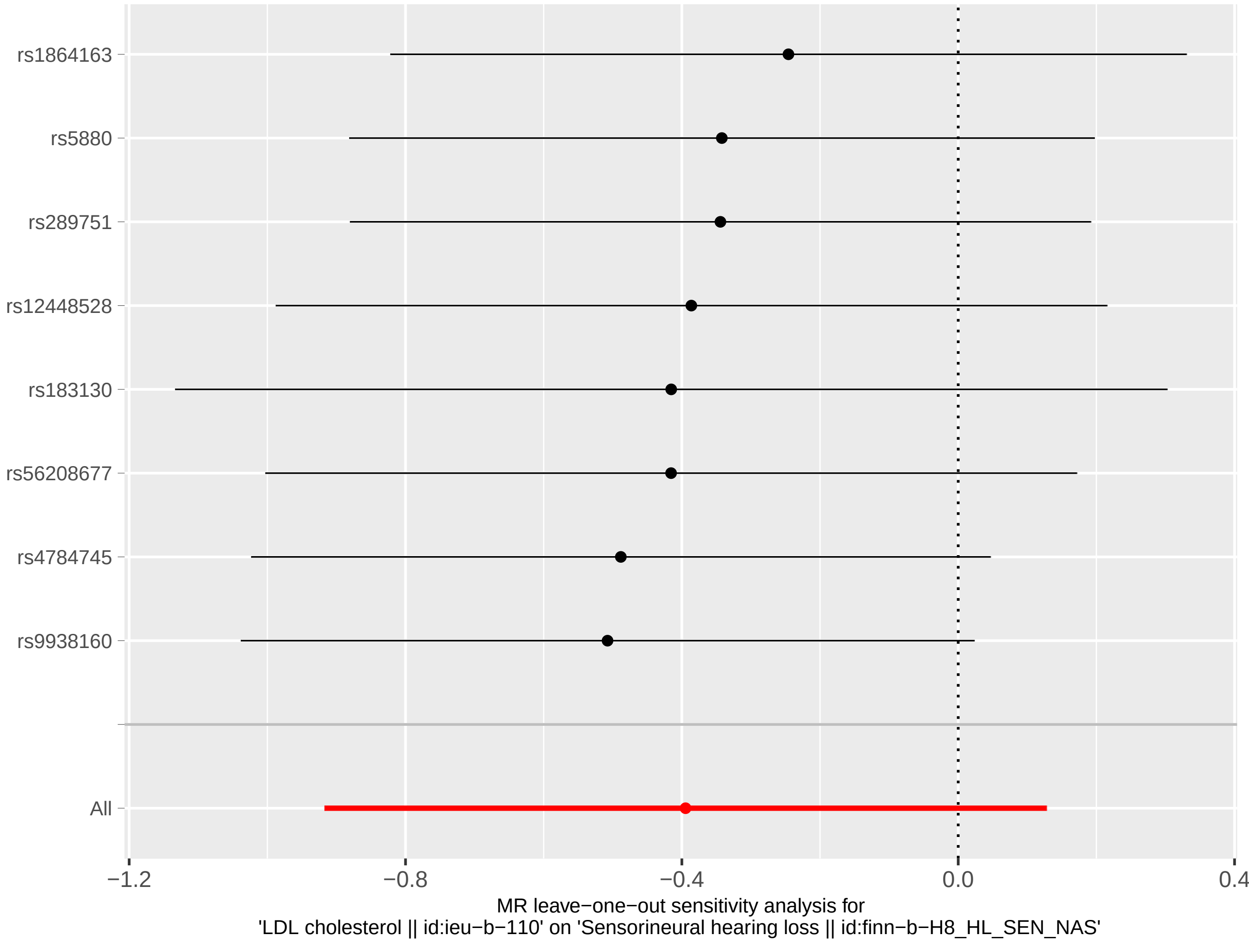
rs1864163
rs5880
rs289751
rs12448528
rs183130
rs56208677
rs4784745
rs9938160
All
−1.2
−0.8
−0.4
0.0
0.4
MR leave−one−out sensitivity analysis for
'LDL cholesterol || id:ieu−b−110' on 'Sensorineural hearing loss || id:finn−b−H8_HL_SEN_NAS'

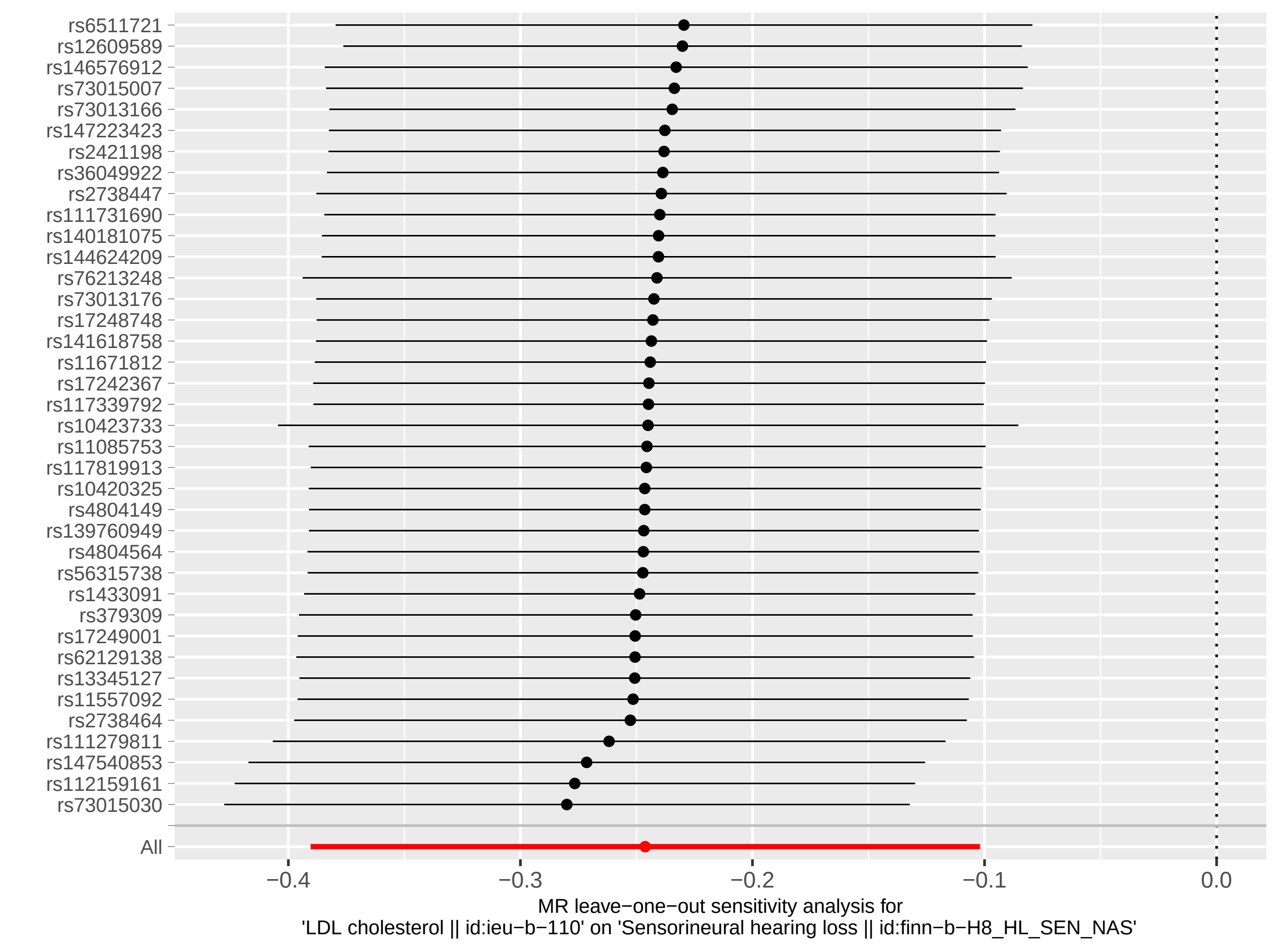
rs6511721
rs12609589
rs146576912
rs73015007
rs73013166
rs147223423
rs2421198
rs36049922
rs2738447
rs111731690
rs140181075
rs144624209
rs76213248
rs73013176
rs17248748
rs141618758
rs11671812
rs17242367
rs117339792
rs10423733
rs11085753
rs117819913
rs10420325
rs4804149
rs139760949
rs4804564
rs56315738
rs1433091
rs379309
rs17249001
rs62129138
rs13345127
rs11557092
rs2738464
rs111279811
rs147540853
rs112159161
rs73015030
All
−0.4
−0.3
−0.2
−0.1
0.0
MR leave−one−out sensitivity analysis for
'LDL cholesterol || id:ieu−b−110' on 'Sensorineural hearing loss || id:finn−b−H8_HL_SEN_NAS'

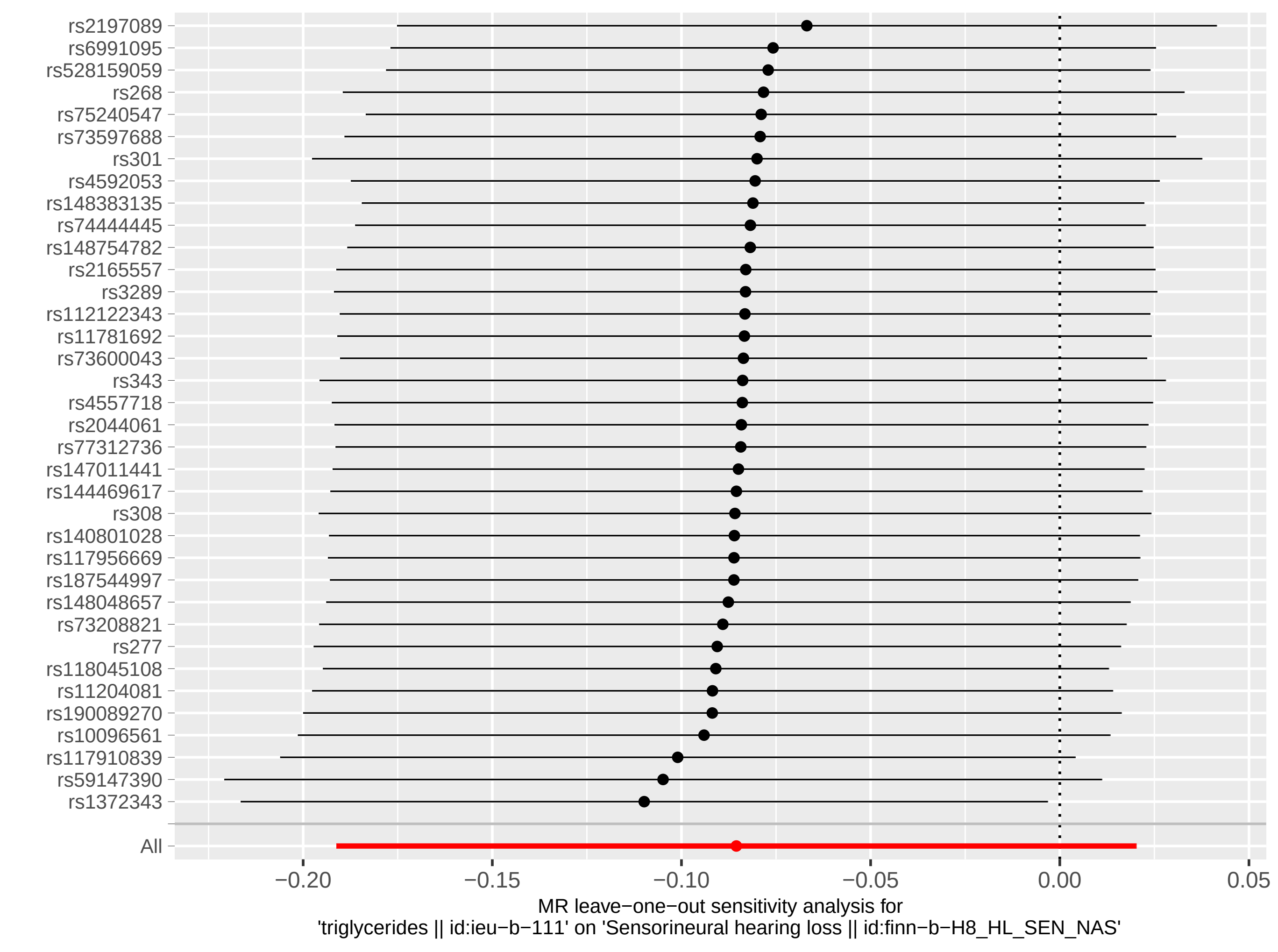
rs2197089
rs6991095
rs528159059
rs268
rs75240547
rs73597688
rs301
rs4592053
rs148383135
rs74444445
rs148754782
rs2165557
rs3289
rs112122343
rs11781692
rs73600043
rs343
rs4557718
rs2044061
rs77312736
rs147011441
rs144469617
rs308
rs140801028
rs117956669
rs187544997
rs148048657
rs73208821
rs277
rs118045108
rs11204081
rs190089270
rs10096561
rs117910839
rs59147390
rs1372343
All
−0.20
−0.15
−0.10
−0.05
0.00
0.05
MR leave−one−out sensitivity analysis for
'triglycerides || id:ieu−b−111' on 'Sensorineural hearing loss || id:finn−b−H8_HL_SEN_NAS'

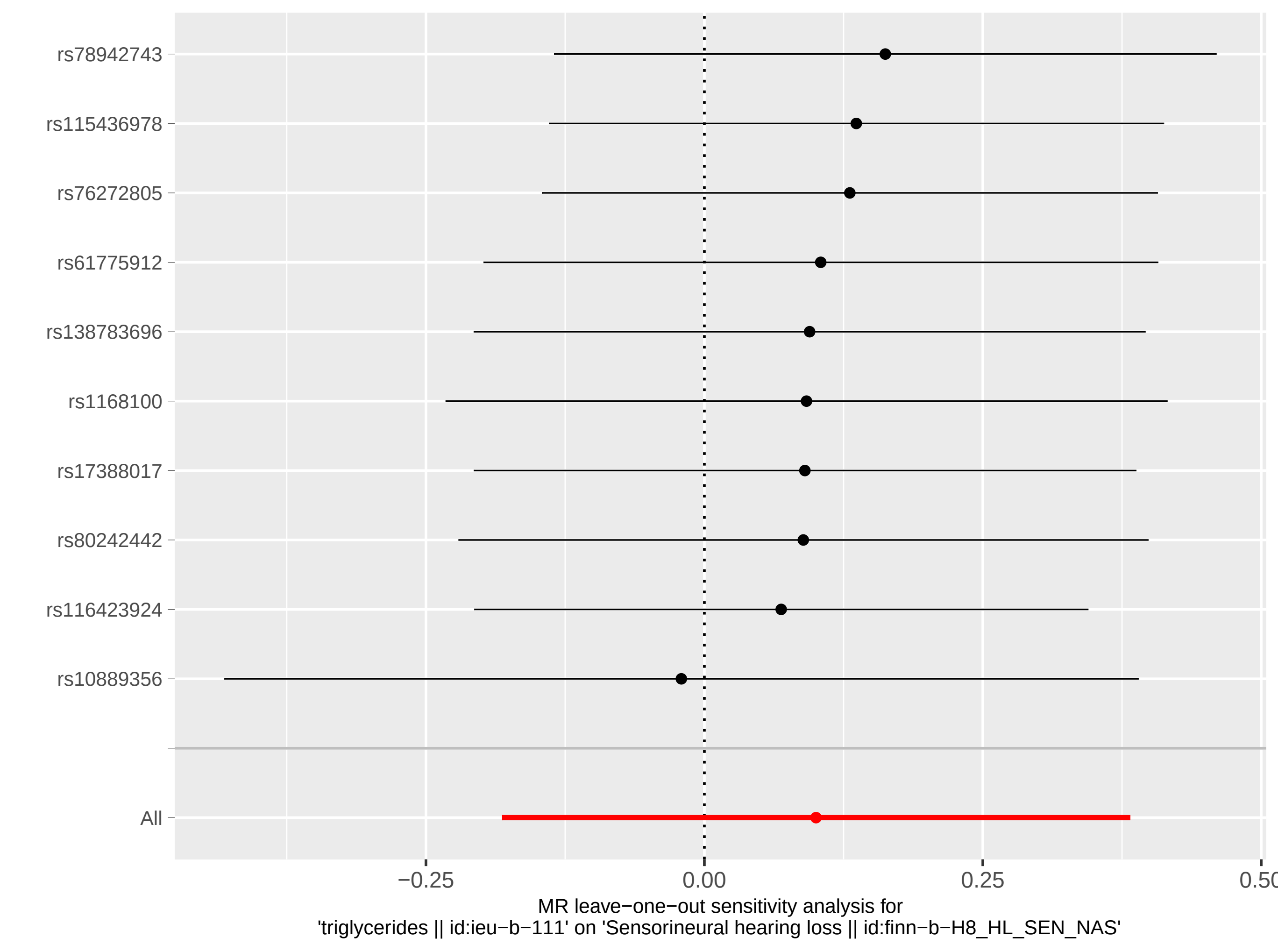
rs78942743
rs115436978
rs76272805
rs61775912
rs138783696
rs1168100
rs17388017
rs80242442
rs116423924
rs10889356
All
−0.25
0.00
0.25
0.50
MR leave−one−out sensitivity analysis for
'triglycerides || id:ieu−b−111' on 'Sensorineural hearing loss || id:finn−b−H8_HL_SEN_NAS'

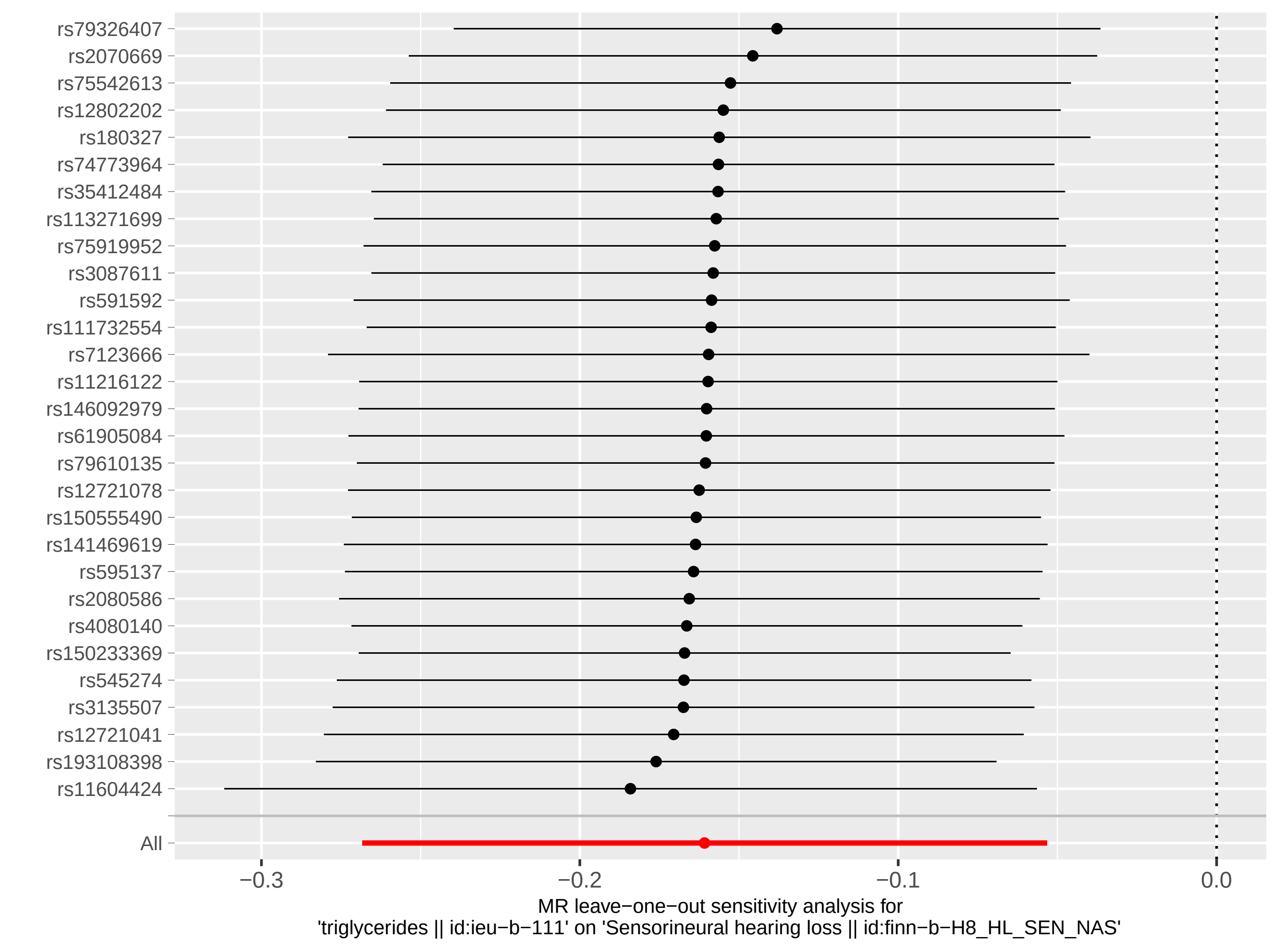

rs79326407
rs2070669
rs75542613
rs12802202
rs180327
rs74773964
rs35412484
rs113271699
rs75919952
rs3087611
rs591592
rs111732554
rs7123666
rs11216122
rs146092979
rs61905084
rs79610135
rs12721078
rs150555490
rs141469619
rs595137
rs2080586
rs4080140
rs150233369
rs545274
rs3135507
rs12721041
rs193108398
rs11604424
All
−0.3
−0.2
−0.1
0.0
MR leave−one−out sensitivity analysis for
'triglycerides || id:ieu−b−111' on 'Sensorineural hearing loss || id:finn−b−H8_HL_SEN_NAS'
